# Mimicking Cellular Membranes With Micropatterned Lipid Bilayers

**DOI:** 10.1002/cphc.70534

**Published:** 2026-08-21

**Authors:** Thuy An Nguyen, Zexi Xu, Petra Schwille

**Affiliations:** ^1^ Department of Cellular and Molecular Biophysics Max Planck Institute of Biochemistry Martinsried Germany

**Keywords:** cellular membrane, microfabrication, micropatterning, supported lipid bilayer, synthetic biology

## Abstract

Studying the role of membranes in cell biophysics requires experimental models which faithfully capture the compositional and topological complexity of cellular structures. This is particularly important for membrane structures that are just at the limit of, or even below optical resolution, such as bacterial cells. Micropatterning is one of the key enabling technologies for the precise manipulation of compositional heterogeneity, morphology, and topography in artificial membranes, bringing in vitro models closer to cellular reality. This perspective highlights recent works which implement micropatterning to mimic cellular membranes and discuss how cellular mechanisms, specifically bacterial ones, may be addressed through the replication of membrane heterogeneity, confinement, and nano‐ and microscale topographical features. It spotlights micropatterning as an invaluable tool for quantitative studies to dissect the topological determinants of cellular function. A thorough understanding of these will lay the foundation for increasingly complex systems to be reconstructed in vitro.

## Graphical abstract

This Perspective spotlights micropatterning of as a key tool to model membrane systems which capture the complexities of cellular membranes, specifically of bacterial ones. Through precise manipulation of compositional heterogeneity, morphology, and topography in artificial bilayers, these synthetic membranes serve as in vitro analogs of cellular membranes, enabling the dissection of various subcellular systems and their molecular determinants.

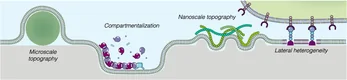

## 1 Introduction

Bacteria, among the simplest organisms found in nature, are excellent specimens for elucidating fundamental mechanisms of cellular function which are otherwise obscured by the complexity of higher organisms. The subcellular details of small bacterial cells, however, have long remained out of reach for light microscopy, which has otherwise greatly contributed to elucidating the inner workings of cells and tremendously benefited cell biology in the past decades. Since the introduction and widespread use of super‐resolution microscopy, the subcellular details of bacteria have begun to come unveiled [1]. In order to back these up with quantitative mechanistic studies, there is an increased need for model systems that can be used as in vitro analogs of the bacterial environment, and of particular importance among these are models of cellular membranes.

Heterogeneous cell membranes and complex protein assemblies within play key roles in a myriad of intra‐ and intercellular processes across multiple spatial and temporal scales. In bacteria, their relevance is further emphasized by the cell‘s smaller dimensions, higher surface‐to‐volume ratio, stronger membrane curvatures, and tight coupling of the membrane to the cell envelope and cytoskeletal structures. A bacterial model should capture these complexities which make the prokaryote’s membrane markedly different from that of eukaryotes. A model which is developed against a certain bacterial species should also replicate the particularities of the organism’s membrane, such as its lipid species, compositional asymmetry and heterogeneities, or its topographies down to the molecular level. This calls for model systems that, beyond general mimicry, can capture the finer details of the membranes’ compositional and topological complexities.

The supported lipid bilayer has become perhaps the most ubiquitous membrane model since Tamm and McConnell first showed that lipid vesicles could rupture and fuse into continuous, fluid bilayers on solid surfaces [2]. It maintains many key properties of biological membranes, including fluidity; can be functionalized with a wide array of biomolecules using various bioconjugation methods; and is compatible with numerous surface‐specific techniques that enable multifaceted insights into the membrane’s structure, composition, mechanical properties, diffusion, and binding kinetics [3, 4, 5, 6, 7]. Yet, the plain supported bilayer lacks many key features of the native membrane’s structure, such as heterogeneity, morphology, or topography. In this context, micropatterning techniques, originating from the material sciences for the fabrication of micro‐ and nanomaterials, have become prevalent in biology. They enable the positioning of proteins for cell adhesion, reshaping of cells in cytoskeletal studies, reorganization of neuronal networks for polarity investigations, and structuring of tissues for unraveling morphogenesis [8, 9]. Micropatterning of membranes was first demonstrated in Groves et al.’s seminal work, where arrays of spatially addressable, chemically distinct lipid bilayer “corrals” were assembled on photolithographically fabricated substrates [10]. Over the past years, micropatterning has given rise to a repository of model membranes that can mimic the spatial heterogeneity of cellular membranes, which presents the focus of this article.

In the following, we outline recent advances which have been enabled by the use of micropatterning on synthetic bilayers. The emphasis lies on how features of cellular membranes can be replicated in these in vitro analogs and how these methodologies could open up new perspectives for bacterial research. Nonbacterial models are discussed as methodological precedents which may be extended to studies of bacterial systems. In this context, we discuss the roles and limitations of micropatterned bilayers in reconstructing increasingly complex membrane systems in vitro.

**FIGURE 1:**
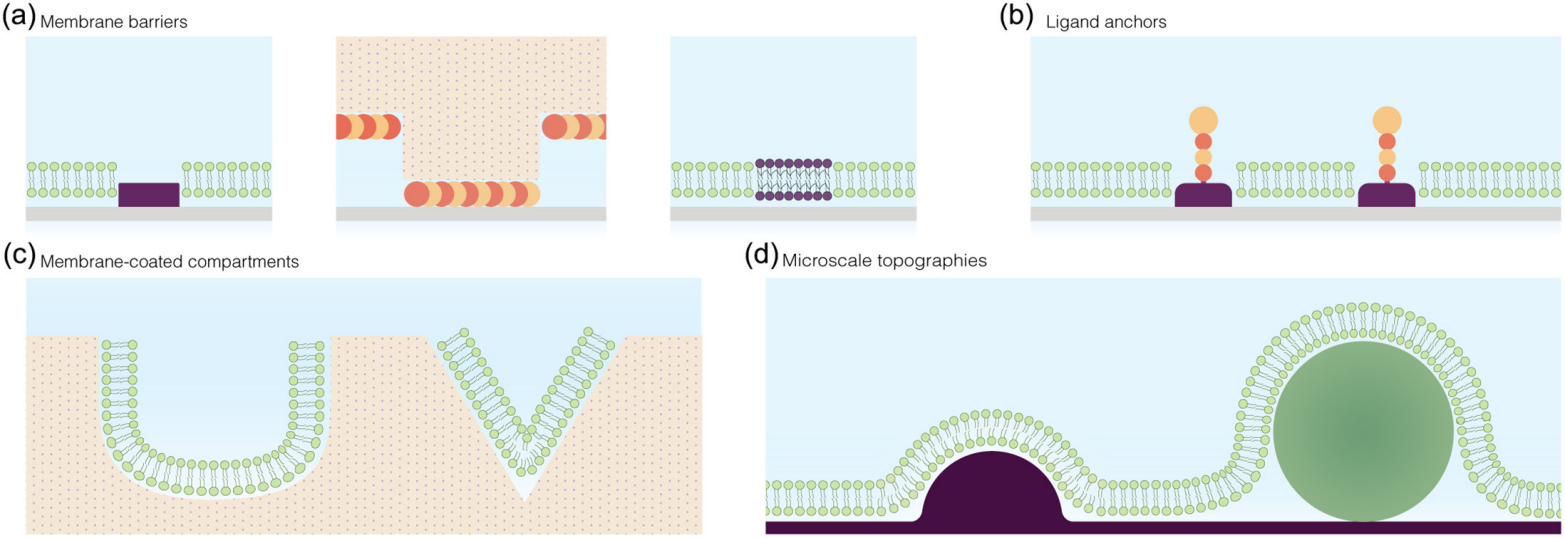
Modifications of synthetic membranes by micropatterning. (a) Physical barriers (purple) to membrane diffusion can be fabricated by lithography (left); microcontact printing, e.g., of a protein (orange) (middle); or photoinduced polymerization of lipids (purple) (right). Barriers enable confinement and can separate compositionally distinct membrane patches. (b) Patterns of ligands or ligand clusters can be achieved through anchoring the ligand (orange) to microfabricated “islands” (purple) within a fluid bilayer. (c) Three‐dimensional membrane compartments of defined geometries can be created by coating a microchamber with a lipid bilayer. (d) Micro‐ and nanoscale topographical features can be achieved through bilayer formation on lithographically fabricated nano‐ and microstructures (purple) or scaffolds (green).

The technical aspects of micropatterning have been extensively reviewed elsewhere [11, 12, 13]. Figure 1 and Table 1 summarize the key concepts, methodological considerations, and representative applications discussed in this Perspective, together with references to relevant methodological reviews and selected use cases.

**TABLE 1:** Replication of cellular membrane features by micropatterning techniques.

Feature of interest	Micropatterning technique(s)	Substrate(s)	Characteristic scale	Accessibility^a^
Membrane domains [14, 15, 16, 17]	Microcontact printing [18] Polymer stencil lift‐off [18] Photopatterning of polymerizable lipids [19]	Glass Polymer‐coated glass	1–10 µm	Moderate‐to‐high accessibility
Ligand patterns [20, 21, 22]	Electron‐beam lithography [23] Photolithography [13] Microcontact printing [18]	Glass	10 nm–1 µm	Limited‐to‐moderate accessibility
Compartments with defined morphologies (two‐ and three‐dimensional) [24, 25, 26, 27, 28]	Photolithography [13] microfluidics	Glass PDMS	10–100 µm	Limited‐to‐moderate accessibility
Curvature [29, 30]	Photolithography [13] Colloidal scaffolding	Glass Colloidal particles	10 nm–1 µm	Limited accessibility
Nano and microscale architectures (pillars, grooves, ridges, complex structures) [31, 32, 33, 34]	Electron‐beam lithography [23] Two‐photon lithography [35] Photolithography [13]	Quartz Photoresist PDMS Glass	100 nm–1 µm	Limited accessibility

a High accessibility: Requires standard laboratory equipment. Moderate accessibility: Requires microfluidics or surface functionalization techniques. Limited accessibility: Requires microfabrication facilities.

## 2 Mimicking Membrane Heterogeneity

Biological membranes are laterally heterogeneous across multiple hierarchical scales, from nanoscale clusters and domains to microscopic cytoskeleton‐confined compartments. Heterogeneous structures in membranes have received great attention with regard to their roles in regulating key processes such as membrane trafficking, junction formation, or signal transduction, the mechanisms of which would be impossible to understand without considering the roles of sterol‐ and sphingolipid‐enriched rafts or the clustering of receptors and signal transducers [36, 37, 38]. Heterogeneity, however, cannot be replicated with homogeneous bilayers as introduced by Tamm and McConnell. To study processes which rely on the subtleties of lateral membrane structure, it is essential to add micropatterned synthetic bilayers to the model systems portfolio.

A such category of lateral heterogeneities are domains which arise from preferential interactions between different lipid moieties in multicomponent membranes. Among these, few are as contentious as the concept of rafts, transient nanoscopic assemblies often enriched in sterol‐ and sphingolipids which are distinct from their surrounding membrane in structure and physicochemical properties [36]. While lipid domains which mimic rafts in vitro form readily in synthetic bilayers given the right composition and under the right conditions [39], many have sought to control the sites of raft mimicries with various approaches [14, 15, 40]. A micropatterned membrane which localizes domain sites can allow for observation and kinetic monitoring of the coalescence of nanoscale cholesterol‐rich structures into microscale domains [41, 42]. Such micropatterns also allow probing isolated interactions of proteins with membrane domains in vitro [16]. Micropatterned membranes can also mimic the pinning of cell membranes by the cortical cytoskeleton and allow the study of membrane‐modulated interactions on a minimal platform [17, 43]. Complementary to these supported membranes are pore‐spanning lipid bilayers, microscale membranes which are decoupled from a solid substrate [44, 45] and whose dimensions are shaped instead by their supporting porous mesh. They enable the study of systems which rely, e.g., on transmembrane processes [46] and a micropatterned porous support allows for precisely defined, suspended membranes on which sterol‐enriched domains can be monitored and analyzed [47]. Suspended membranes thus broaden the capabilities of micropatterning from structuring the support to modulating the boundary conditions; together, these approaches could offer new perspectives on the microdomains of prokaryotic membranes, which have been studied in vivo but, unlike their eukaryotic counterparts, are yet to be dissected in vitro [48]. As the separation of, e.g., cardiolipin‐enriched domains from an initially homogeneous membrane is inherently stochastic, the ability to determine the site of these domains would allow researchers to closely follow and unravel the molecular mechanisms underpinning domain assembly and their interactions with other bacterial subcellular systems.

Living membranes undergo further heterogeneous restructuring during signaling processes, when clusters of receptors and signal transducers nucleate and coarsen at the cell’s signaling interface. In eukaryotes, signaling interfaces such as the immunological synapse between a T cell and an antigen‐presenting cell or E‐cadherin adhesions and ephrinA1–EphA2 complexes at the junction of epithelial cells are frequently investigated using hybrid platforms consisting of live cells and a synthetic bilayer, but the lack of spatiotemporal control on these platforms often hinder more detailed mechanistic investigations. Here, micropatterning has enabled cluster patterns to be replicated for such studies through fabrication of nano‐ and microscale structures that anchor specific ligands within a supported membrane [20, 21, 49, 50]. The triggering of T cell receptors by the peptide‐major histocompatibility complex (pMHC) ligand has been investigated in this way, allowing dissection of molecular details down to the spacing, packing, and axial positioning of ligands for optimal T cell response (Figure 2a) [20]. Besides precise ligand positioning, micropatterning also enables spatial perturbations of signaling interfaces by the placement of diffusion barriers, through which the molecular mechanisms underpinning the activation of signaling can also be examined [22, 54, 55, 56]. These methodologies have mainly been applied to eukaryotic systems but could conceivably be extended to prokaryotic signaling processes. The mimicry of membrane heterogeneity by micropatterning, however, has its limitations. The multiscale domains and clusters of living cells are not static, but rather emerge, persist, and dissolve through active cellular processes, while microfabricated domains and clusters are rigid, preimposed structures. These platforms are not replicas of living membranes; rather, they enable the isolation and detailed interrogation of variables which contribute to membrane behavior. They are complementary to live‐cell methods which enable observation of native membrane dynamics, including lipid turnover, cytoskeletal pinning, clustering, transport, and remodeling, but where simultaneous processes obscure the link between a phenotype and single variables.

FIGURE 2Micropatterned membranes enable the in vitro dissection of cellular systems. (a) Nanopatterned arrays of peptide‐major histocompatibility complexes trigger the T cell signaling cascade. Scanning electron micrographs of nanoarrays and fluorescence images of the resulting signalosome pattern (green) of activated T cells. Different sizes of ligand clusters result in different signalosome intensities. Scale bar: 5 μm. Reproduced with permission from [20]. Copyright 2018, Springer Nature. (b) Two‐dimensional geometry sensitivity of *E. coli* Min proteins on geometrically defined membrane corrals. The propagation direction of Min protein waves consistently aligns with the longest axis of the membrane patch. Scale bar: 25 μm. Reproduced with permission from [25]. Copyright 2012, National Academy of Sciences. (c) Reconstitution of the Min system and the bacterial tubulin‐homologue FtsZ in membrane‐coated polymer microchambers. (Left) FtsZ (green) is positioned at the “mid‐cell” of the rod‐shaped compartment Min oscillations, with labeled MinE (magenta). Time between frames: 20 s. (Right) Time‐averaged gradient of FtsZ‐MTS and the Min system and schematic illustration of FtsZ positioning in membrane microcompartment. Scale bar: 5 μm. Adapted from [51] under the CC BY 4.0 license. (d) Reversible deformation of liposomes containing FtsZ (green) in microfluidic traps under constant osmolarity. FtsZ transitions between rings and elongated filaments, dependent on the liposome shape. Scale bar: 10 μm. Adapted from [28] under the CC BY 4.0 license. (e) FtsZ filaments align with curved membranes, indicating an intrinsic filament curvature that matches that of the membrane. (Left) Alignment of MTS‐FtsZ‐YFP on curved membranes atop glass capillaries with regards to the capillary axis, dependent on membrane curvature. (Right) FtsZ (green) bound to curved membranes of various radii. Scale bar: 1 µm. Adapted with permission from [29]. Copyright 2012 WILEY‐VCH Verlag GmbH & Co. KGaA, Weinheim. (f) Patterns of MinD (green) and MinE (unlabeled) propagate on a membrane‐coated (magenta) nanofabricated three‐dimensional structure, guided by topographical cues. Topography influences the patterns of Min oscillations. Scale bar: 100 µm. Adapted from [34] under the CC BY‐NC 3.0 license. (g) Dissecting the effects of FtsZ‐induced torsional stress on membranes with microfabricated conical structures. FtsZ rings (green) form dynamic screw‐like vortices inside conical microstructures coated with a lipid bilayer (magenta), mimicking the conical inward deformations induced by FtsZ in liposome membranes. Reproduced from [52] under the CC BY 4.0 license. (h) The organization of linear cortical actin responds to topographical cues. A liposome containing mDia1‐nucleated linear cortical actin adhering to patterned microridges. The linear actin cortex aligns itself with the ridge axis. Adapted with permission from [53]. Copyright 2024, Elsevier.

**Figure:**
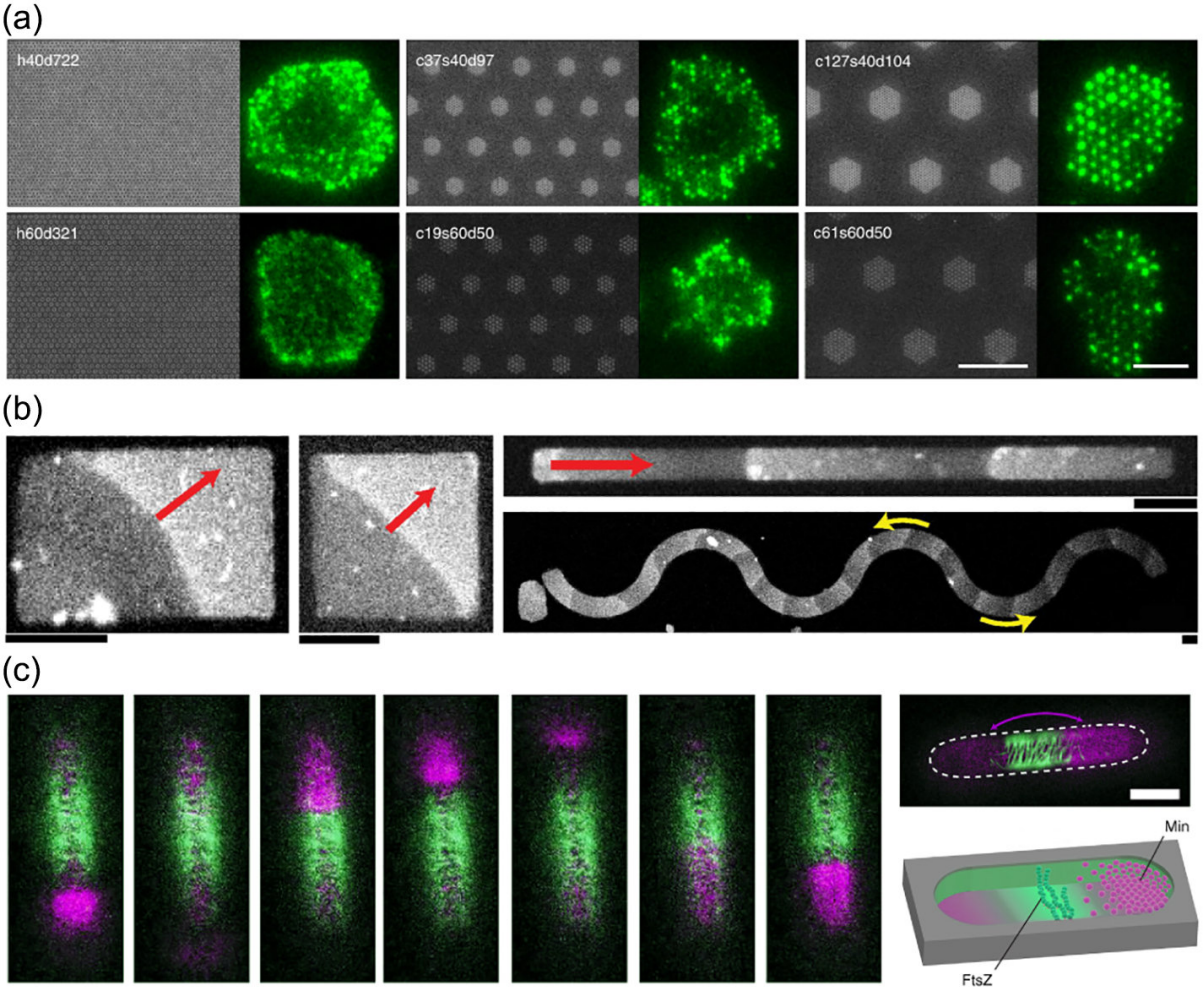

**Figure:**
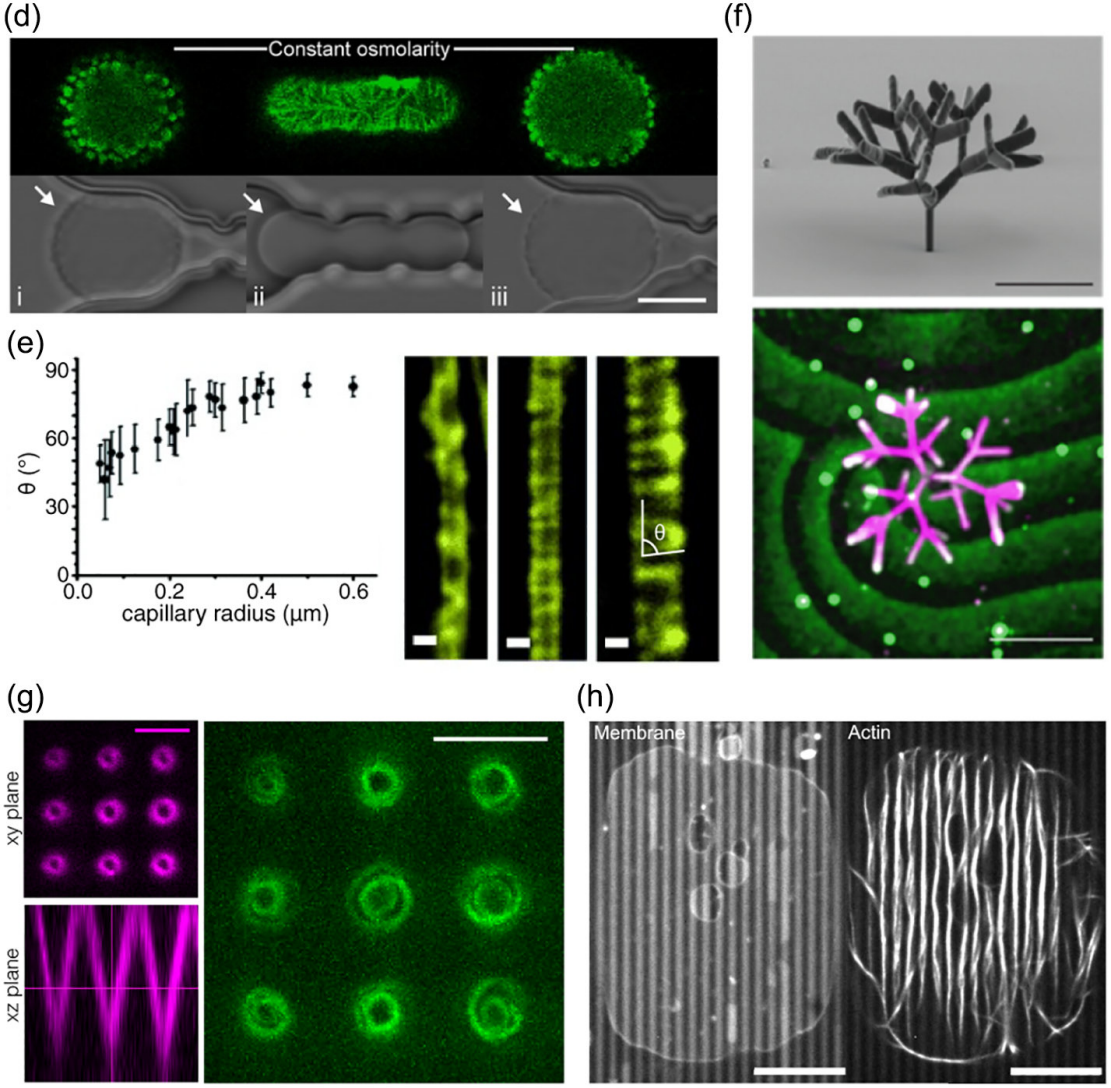

## 3 Modeling 2D and 3D Compartmentalization

Molecular interactions which are confined to the surface of membranes fundamentally differ from those occurring in the cytosol or extracellular space, as the two‐dimensionality of the membrane modulates the diffusion and interactions kinetics of its molecules [3]. How compartmentalization of this surface affects molecular interactions remain elusive in homogeneous and practically infinite planar membranes, but they can be modeled by micropatterned bilayers.

This question has been investigated for the eukaryotic phosphoinositide lipids (PIP) or the bacterial Min proteins, self‐organizing systems in which spatial gradients play key regulatory roles in cellular processes. When reconstituted on micropatterned membrane arrays of varying patch sizes, the PIP kinase–phosphatase system revealed a bistability switch dictated by confinement size, resulting in compositional patterns on larger membrane patches through stochastic kinase–phosphatase competition and more homogeneous distributions on smaller ones [24, 57]. *E. coli* Min proteins, when reconstituted in micropatterned membranes of varying dimension ratios, self‐organized into traveling waves that align with the longest confinement axis and could synchronize across adjacent patches (Figure 2b) [25]. The self‐assembly of cytoskeletal structures, such as actin, a system which extends into three‐dimensional networks but nucleates on the lipid membrane, is also impacted by spatial confinement [26, 58]. Constraining actin nucleation‐promoting factors to micropatterned bilayer patches revealed the role of membrane compartmentalization in regulating actin network architecture [26]. At the single‐molecule level, micropatterned confinement has also been used to unravel the molecular mechanisms of individual signaling molecules in a crowded membrane environment [59, 60].

How does compartmentalization in the third dimension affect molecular interactions on a cellular scale? Beyond the two‐dimensionality of the membrane surface, molecular interactions at and with the membrane also respond to the cell’s shape, the membrane’s curvature, the presence of protrusions or invaginations, and interactions between the cell and a surface. Such compartmentalization‐dependent behavior can be observed in living cells, but active processes in vivo make it difficult to determine whether one of these variables alone is responsible for the observed response. These parameters thus must be recapitulated in vitro, by extending micropatterning to three‐dimensional architectures.

In *E. coli*, the Min system positions the division site through pole‐to‐pole oscillations that are sensitive to geometry, with distinct oscillatory modes arising under different boundary conditions [61, 62, 63, 64]. At larger scales, phytoplankton undergo rapid cytoskeleton‐associated transitions from asymmetric to more symmetric morphologies in response to hydrodynamic cues [65]. These examples underpin two fundamental phenomena: how bulk–surface molecular systems respond to compartmentalization in three dimensions and how cytoskeletal assemblies behave differently when constrained by deformable versus rigid boundaries, potentially reshaping those boundaries in return. These questions have been addressed in vitro with the microfabrication of three‐dimensional membrane systems [27, 28, 51, 54, 66, 67, 68]. Membrane‐coated microchambers of rod‐like geometries show that the oscillatory modes of Min proteins depend on and strongly respond to boundary conditions (Figure 2c) [51, 66]. Liposomes reshaped by adhesion patterns allow investigations of how cortical actin responses to compartmentalization [53]. The reshaping of liposomes can also be made reversible with microfluidic technologies, through which the topology‐dependent arrangement and rearrangement of FtsZ‐based cytoskeletal structures have been dissected (Figure 2d) [27, 28, 68]. Together, these advances chart a trajectory from open‐well microchambers to enclosed, shape‐tunable bilayers, which reveal how cell‐scale morphology regulates biomolecular assemblies, extending the micropatterning toolbox into the third dimension.

## 4 Replicating Nano‐ and Microscale Topographies

Beyond being a mere consequence of cell shape, topographical features of the membrane itself may act as cues for several cellular processes [69]. These mechanisms are challenging to study in vivo due to the transient character of such processes, and micropatterned bilayers have become valuable in vitro models for membrane topography. By fabricating 3D substrates or scaffolds, membrane curvature can be varied independently and the response of lipids, cytoskeletal filaments, or proteins quantified.

Curvature can regulate lateral membrane structure. Curved membranes have been observed to induce lipid sorting, raft formation, and raft‐associated leaflet asymmetry [70, 71], as well as define the preferential binding sites of cytoskeletal and regulatory proteins [72, 73]. Investigations performed on microfabricated curved membranes demonstrated that filaments of the prokaryotic tubulin homolog *E. coli* FtsZ align robustly with membrane curvatures and readily assemble into ring structures in grooves mimicking the radial dimensions of the bacterial cell, highlighting curvature as a determinant of bacterial cytokinesis (Figure 2e) [29]. Curved membranes formed on spherical beads also revealed the preference for low‐curvature regions of SecA, a facilitator of protein translocation in bacteria [30]. Synthetic molecules can also have a curvature preference. Conjugated oligoelectrolytes with antimicrobial activity can intercalate into bilayers at nanobar‐induced curvature sites and remodel the membrane, offering a mechanistic model for how small antimicrobial molecules can compromise bacterial envelopes [74]. Nano and micropatterning, thus, enable curvature to be interrogated across a spectrum relevant for cellular membranes, providing an experimental handle on processes ranging from bacterial cytokinesis to toxin binding.

Topographical cues imposed by the nano‐ and microscale architectures outside the cell also modulate cellular behavior. Nanoscale roughness and ridges have been shown to modulate cytoskeletal structure and membrane trafficking through deforming the membrane of eukaryotic cells [31]. but the influence of such topographical features has been little explored in prokaryotes. The emerging biological question is how microbes engage in cross‐talk with their microenvironment and how topography mediates these interactions [75, 76].

Micro‐ and nanoscale architectures can help isolate the topography dependence of cell‐scale membrane systems. The oscillatory patterns of Min proteins can propagate across complex membrane‐coated microfabricated structures (Figure 2f) [32, 34]. FtsZ assembles into vortices inside conical membrane‐coated microstructures, which resemble the screw‐like deformations it induces in liposome membranes through torsional stress (Figure 2g) [52]. Linear cortical actin filaments align themselves alongmicroscale ridges (Figure 2h) [53], while multivalent protein condensates accumulate at edges, deform within grooves, and migrate along ridges under capillary forces [33]. Together, these studies show that membrane topography can bias the localization, organization, and dynamics of membrane‐associated systems. In living cells, however, topographical sensing is coupled to active cytoskeletal feedback, membrane tension regulation, trafficking, and protein turnover, which can obscure the specific contribution of topography. Micropatterned membranes therefore provide a complementary route to dissect how topographical cues are sensed, translated into molecular organization, and potentially reinforced through feedback.

## 5 Conclusion and Perspectives

The introduction of micropatterning methodologies to cell membrane mimicries has given rise to a wide repository of biomimetic platforms on which many features of the native membrane, including compositional heterogeneities, compartmentalization, morphology, and topographies have been replicated and studied in detail. These membrane features span multiple length scales, from nanoscale lipid domains and receptor clusters, submicron cytoskeleton‐confined compartments, protrusions and invaginations, to cell‐scale boundaries. The biological interpretation of micropatterning experiments therefore depends on whether the selected dimensions are matched to the process under study. Compartmentalization changes the effective length scale over which molecular diffusion and reaction occur [3, 24], and the compartment’s form and dimensions can alter the behavior of clusters, filaments, and oscillatory patterns [24, 25, 26, 51, 54, 62]. Nano‐ and microscale topographies guide the preferential binding of regulatory proteins and filamenting structures along curves, grooves, and ridges [29, 30, 52, 53]. Recent advances, from nanoscale lithographic patterning to three‐dimensional membrane‐coated architectures, now make it possible to tune these parameters across multiple relevant length scales [13, 20, 23, 34, 35]. A key direction could be multiscale methodologies to enable more sophisticated in vitro platforms which could probe interactions across scales and, potentially, any feedback between them, mechanisms that would remain elusive in current models.

In the larger scheme, the micropatterning of in vitro membranes and the micropatterning of live cells occupy complementary positions, along a trade‐off between experimental control and physiological complexity. Synthetic platforms allow the variables of membrane structure to be independently and systematically controlled, whereas live‐cell micropatterning preserves cytoskeletal coupling, active remodeling, trafficking, protein turnover, and complex protein–lipid interactions [8, 62]. Collectively, these advances bridge the gap between simplistic models and the complex conditions of biological systems. Yet it remains a colossal challenge to reproduce the complete three‐dimensional architecture of a cell’s membranes, its full spectrum of structural particularities, and raveled functionalities. This tremendous task could not be accomplished without a comprehensive and ever‐improving toolbox, which includes techniques to manipulate membranes, as referred to in this article, but also those that can manipulate biomolecules, synthetic structures, and living organisms, such as micropatterning cellular structures and whole cells [62, 77, 78], alongside those that can control their microenvironment. In this regard, focus should be placed on the reconstitution of larger cellular structures and machineries on membrane platforms, e.g., the functional integration of transmembrane proteins with bulky cytosolic and extracellular domains.

Micropatterned membranes are promising for the elucidation of physical principles that govern key functions in bacterial cells. A key opportunity is the use of micropatterning to mimic bacterial functional membrane microdomains reminiscent of rafts in eukaryotic cells, providing insights on how such domains could interact with other bacterial systems in vivo and shedding light on the fundamental principles of bacterial membrane organization [38, 79, 80, 81]. Similarly, micropatterned membranes could be good models of host membranes encountered by bacteria during infection, enabling mechanistic dissection of host–pathogen interactions under controlled conditions [82, 83]. In addition, scaffolded and sculpted membranes may become essential for understanding how bacterial proteins interpret topography, allowing, e.g., investigations of whether proteins such as *E. coli* MinD and FtsZ act as curvature sensors, generators, or both. These questions remain largely unexplored in bacterial systems and can be extended to other microorganisms, and micropatterned membranes are a compelling, if speculative, approach for finding their answers.

## Funding

This study was supported by the Deutsche Forschungsgemeinschaft (521256690), Max Planck‐Bristol Centre for Minimal Biology, and International Max Planck Research School for Molecules of Life.

## Conflicts of Interest

The authors declare no conflicts of interest.

## Disclaimer

The opinions expressed in this publication are the view of the author(s) and do not necessarily reflect the opinions or views of ChemPhysChem, the Publisher, ChemistryEurope, or the affiliated editors.

## Data Availability

Data sharing not applicable to this article as no datasets were generated or analyzed during the current study.
